# Artificial intelligence assisted quantitative analysis of fundus structural and vascular alterations reveals fundus tessellation as a robust biomarker of myopia severity

**DOI:** 10.3389/fcell.2026.1860617

**Published:** 2026-06-30

**Authors:** Luxiang Sun, Dongqing Yuan, Chenfeng Gu, Bin Lou, Hong Cheng

**Affiliations:** 1 School of Basic Medical Sciences & School of Public Health, Faculty of Medicine, Yangzhou University, Yangzhou, China; 2 Department of Ophthalmology, The First Affiliated Hospital with Nanjing Medical University, Nanjing, Jiangsu, China; 3 Department of Ophthalmology, Children’s Hospital of Nanjing Medical University, Nanjing, China

**Keywords:** artificial intelligence, biomarker, fundus tessellation, myopia, oculomics, peripapillary atrophy, retinal vasculature

## Abstract

**Background:**

Myopia is characterized by progressive axial elongation and structural remodeling of the posterior segment, ultimately leading to irreversible visual impairment in high myopia. However, early, noninvasive biomarkers that capture the continuum of fundus alterations across myopia severity remain insufficiently defined. Recent advances in artificial intelligence (AI) enable high-throughput quantitative analysis of fundus images, providing new opportunities for identifying imaging-based biomarkers.

**Methods:**

This cross-sectional observational study included 539 eyes from 274 participants with varying degrees of myopia. Based on cycloplegic spherical equivalent (SE), eyes were categorized into low, moderate, high, and super-high myopia groups. Color fundus photographs were analyzed using an AI-assisted framework to quantify fundus tessellation (FT), peripapillary atrophy (PPA), optic disc morphology, and retinal vascular parameters, including fractal dimensions, vessel geometry, and vessel density. Logistic regression and receiver operating characteristic (ROC) analyses were performed to identify independent biomarkers and evaluate their discriminative performance. Linear regression was used to assess associations with SE.

**Results:**

Fundus structural and vascular parameters showed significant differences across myopia severity groups. FT-related parameters increased progressively with increasing myopia severity (all P < 0.001), with macular FT area demonstrating the strongest association. Retinal vascular complexity and vessel density decreased with increasing myopia severity, reflected by reduced fractal dimensions and vessel density (all P < 0.001). In multivariable analysis, macular FT area (OR = 2.925, P < 0.001) and PPA height (OR = 1.501, P = 0.001) were independently associated with high myopia, while vertical optic cup diameter showed an inverse association (OR = 0.664, P < 0.001). Vascular parameters did not retain independent significance after adjustment. ROC analysis showed that macular FT area achieved the highest discriminative performance (AUC = 0.819), and a combined model yielded an AUC of 0.869. Linear regression demonstrated a strong association between FT parameters and SE.

**Conclusion:**

Fundus structural alterations, particularly fundus tessellation and peripapillary atrophy, are the most robust imaging biomarkers associated with myopia severity. While refraction and axial length remain the primary measures of myopic severity, FT provides complementary information about posterior segment remodeling. Prospective studies are needed to evaluate whether FT predicts future complications such as myopic maculopathy or retinal detachment. Retinal vascular changes appear secondary and contribute less to disease discrimination. AI-assisted quantitative fundus analysis provides a noninvasive and scalable approach for identifying individuals at risk of high myopia and offers new insights into the structural–vascular remodeling underlying myopia progression.

## 1 Introduction

Myopia has become a major global public health concern, with its prevalence projected to affect nearly half of the world’s population by 2050, including approximately 10% with high myopia (Holden et al., 2016). Beyond refractive error, high myopia represents a progressive structural disorder associated with irreversible vision loss due to retinal detachment, myopic maculopathy, glaucoma, and choroidal neovascularization (Ohno-Matsui, 2025; Arnal et al., 2025a; Arnal et al., 2025b).

The hallmark of myopia is axial elongation–induced remodeling of the posterior segment, leading to progressive thinning of the retina and choroid and characteristic fundus changes such as fundus tessellation (FT), peripapillary atrophy (PPA), and optic disc deformation (Jonas et al., 2023; Xu et al., 2023). These structural alterations reflect underlying biomechanical and vascular remodeling processes.

Recent advances in AI have enabled objective quantification of fundus features from color fundus photography (CFP), a widely accessible imaging modality (Guo et al., 2024; Yang et al., 2025). Among these features, FT-representing increased visibility of large choroidal vessels-has emerged as a potential biomarker of choroidal thinning and disease severity. Concurrently, the retinal vascular network exhibits fractal properties, and its complexity can be quantified using fractal dimension (FD), providing insights into microvascular remodeling (Zekavat et al., 2022).

Emerging evidence further suggests that myopia involves dysregulation of the neurovascular unit, including hypoxia-driven signaling pathways such as HIF-1α, as well as inflammatory and growth factor–mediated remodeling processes (Wen et al., 2025; Meng et al., 2025; Mérida et al., 2025). These findings highlight the need for integrated imaging biomarkers that capture both structural and vascular alterations.

However, most prior studies have focused on isolated parameters, lacking a comprehensive, multidimensional analysis using routine imaging (Xu et al., 2025; Qiu et al., 2025). Therefore, this study aimed to systematically quantify FT, optic disc morphology, and retinal vascular features from CFP using AI-assisted analysis, and to identify robust imaging biomarkers associated with myopia severity.

## 2 Materials and methods

### 2.1 General information

This study was designed as a cross-sectional observational study and conducted at Jiangsu Province Hospital between 2023 and 2025. Participants with myopia who underwent routine ophthalmic examinations during the study period were consecutively enrolled. Basic demographic and refractive information were collected for all participants, including sex, age, spherical power, cylindrical power, and spherical equivalent (SE). All subjects underwent cycloplegic refraction, slit-lamp biomicroscopy, fundus examination, and color fundus photography to determine refractive status and to exclude other ocular diseases. Inclusion criteria were: (1) age ≥18 years; (2) cycloplegic SE between −0.50 D and −20.00 D; (3) best-corrected visual acuity (BCVA) of 20/40 or better; and (4) clear fundus images suitable for AI-based segmentation. Exclusion criteria were: (1) astigmatism >2.00 D or anisometropia >1.50 D; (2) previous ocular surgery (including refractive, cataract, or vitreoretinal surgery); (3) presence of any ocular pathology other than myopia (e.g., diabetic retinopathy, age-related macular degeneration, glaucoma, uveitis); (4) history of ocular trauma or laser photocoagulation; and (5) media opacities (e.g., significant cataract or vitreous hemorrhage) precluding clear fundus visualization. Based on cycloplegic SE, eyes were classified into four groups: low myopia, moderate myopia, high myopia, and super-high myopia, according to established refractive criteria (low myopia: spherical equivalent [SE] −0.50D to −3.00D; moderate myopia: SE –3.01D to −6.00D; high myopia: SE –6.01D to −10.00D; super-high myopia: SE ≤ −10.01D). This study adopted an eye-based analytical design, in which all eyes meeting the inclusion criteria were included in the analysis. A total of 539 eyes from 274 eligible participants were finally analyzed. The study protocol adhered to the tenets of the Declaration of Helsinki and was approved by the Institutional Review Board of Jiangsu Province Hospital. Written informed consent was obtained from all participants or their legal guardians prior to enrollment.

### 2.2 Research methods

#### 2.2.1 Color fundus photography and image quality control

Color fundus images were acquired using a CLARUS™ 500 (ZEISS, Dublin, CA, USA) in a darkroom environment. All photographs were captured by a single trained operator to minimize inter-operator variability. For each eye, two 133° color fundus images centered on the optic disc were obtained using a CLARUS™ 500. Although the CLARUS™ 500 acquires 133° widefield images, a standardized 45° field of view centered on the optic disc was extracted from each widefield image for quantitative analysis. Extraction involved automated optic disc detection followed by cropping to a fixed 45° diameter circle centered on the disc center.

#### 2.2.2 Fundus tessellation segmentation and quantification

**FIGURE 1:**
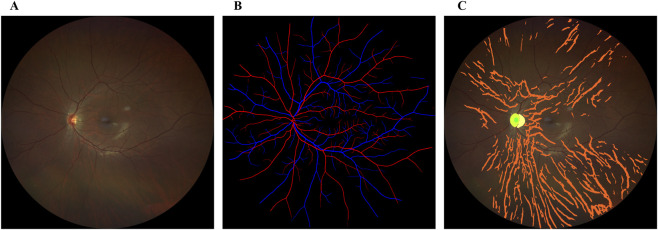
Quantification of Retinal Vascular Parameters from Color Fundus Photography **(A)** Input fundus image **(B)** artery-vein segmentation, with veins represented in blue, arteries in red **(C)** fundus tessellation segmentation, fundus tessellation in orange, peripapillary atrophy in white, optic disc in yellow, optic cup in green.

We employed an automated measurement system for retinal vascular parameters, which leverages AI technology and was previously developed by Jiang et al. (2023). This system was used for the segmentation and quantification of color fundus photographs, as depicted in Figure 1. Fundus tessellation (FT), defined as the visibility of large choroidal vessels at the posterior fundus pole outside the peripapillary region, was segmented using a method adapted from previous studies (Shao et al., 2021; Chen et al., 2023). Specifically, FT regions were manually annotated on color fundus photographs, and a deep learning-based segmentation network was trained using these annotations. The trained network was subsequently applied to segment FT regions in the images included in the present study. All segmentation results were manually reviewed, and minor corrections were applied when necessary to ensure segmentation accuracy. Pixel measurements were converted to physical units using the manufacturer-provided conversion factor (1 pixel = 0.007 mm at the retinal plane for a standard emmetropic eye). Individual magnification correction based on axial length was not applied because axial length was not measured. Consequently, absolute area measurements may be affected by spherical-to-planar projection distortion inherent in 2D fundus photography, as a 3D spherical retina is flattened onto a 2D image plane. In highly myopic eyes with longer axial length, the actual retinal magnification differs from the emmetropic model, leading to systematic overestimation of absolute area measurements. However, because our primary analyses compare relative differences across myopia severity groups and the direction of overestimation is consistent within each group, this limitation does not reverse the observed associations.

FT area was calculated as:

$$\text{Area }\left(\text{FT}\right)=number\text{ of pixels labeled as tessellation}\times \left(\text{pixel}\_\text{spacing}\_x\times \text{pixel}\_\text{spacing}\_y\right)$$

Based on the extracted tessellated area, FT density was calculated to normalize for inter-individual differences in fundus size:

$$\text{Density }\left(\text{FT}\right)=\text{Area }\left(\text{FT}\right)/\text{Total Fundus Area}$$

The total fundus area was obtained by applying thresholding and morphological filtering to remove the black background region.

In addition to mean FT density and total FT area, the following FT- and peripapillary-related parameters were extracted: mean FT density, total FT area, macular FT area, peripapillary FT area, peripapillary atrophy-to-disc area ratio (PPA/D area ratio), peripapillary atrophy width and height, horizontal and vertical PPA-to-disc diameter ratios. Quantitative assessment of FT provides an objective biomarker reflecting choroidal and retinal structural alterations associated with myopia.

#### 2.2.3 Quantitative analysis of optic disc, optic cup, and retinal vessels from color fundus photographs

Quantitative analysis of optic disc, optic cup, and retinal vascular parameters was performed following established analytical frameworks described by Chen Q et al. (2025). Optic disc, optic cup, retinal arteries, and retinal veins were segmented using a high-resolution convolutional neural network (HR-Net), which preserves fine semantic and spatial features and is suitable for precise anatomical segmentation. After optic disc segmentation, the retinal region was divided into three concentric zones based on the distance from the optic disc margin, expressed in disc diameters (DD): Zone A: 0–0.5 DD; Zone B: 0.5–1.0 DD; Zone C: 1.0–2.0 DD.

Retinal arteries and veins were subsequently segmented using HR-Net (Cao et al., 2025). The resulting vessel masks were skeletonized using the Zhang-Suen thinning algorithm to extract one-pixel-wide centerlines (Li et al., 2023). Vascular bifurcation points were identified by applying morphological operations to the extracted centerlines.

#### 2.2.4 Fractal dimension analysis from color fundus photographs

Four fractal dimensions were calculated separately for retinal arteries and veins using the vessel centerlines extracted from color fundus photographs: capacity dimension (D_0_), entropy dimension (D_1_), correlation dimension (D_2_), and singularity length (SL). Fractal dimensions were calculated using the vessel centerlines as input, applying the box-counting method implemented in the validated Public Vascular Biometrics Model (PVBM) toolbox (Fhima et al., 2023). The box-counting method involves overlaying a grid of varying box sizes onto the vessel skeleton and counting the number of boxes containing vessel pixels. The fractal dimension is derived from the slope of the log-log plot of box count versus box size. Higher fractal dimension values indicate greater vascular network complexity and space-filling capacity.

#### 2.2.5 Vascular branching and tortuosity parameters from color fundus photographs

In Zone C, arterial and venous branching parameters were quantified according to the definitions proposed by Sun et al., including: average branching angle (angle_avg), average branching asymmetry (asymmetry_avg), and average number of branches (branch_avg) (Sun et al., 2022). Additional vascular geometric parameters were calculated, including: average vessel length (length_avg), average tortuosity (curvature_avg), and vessel length density (vessel_length_density). Curvature_avg was defined as the ratio of the actual centerline length of a vessel segment to the straight-line distance between its endpoints, with lower values indicating straighter vessels.

These parameters capture distinct aspects of vascular network architecture: branching angle reflects the geometry of bifurcations, tortuosity indicates vessel curvature, and vessel_length_density represents the total vessel length per unit area (Zheng et al., 2025; Gong et al., 2024).

#### 2.2.6 CRAE, CRVE, and AVR from color fundus photographs

Central retinal arteriolar equivalent (CRAE) and central retinal venular equivalent (CRVE) were calculated using the revised Knudtson formula based on vessel caliber measurements from color fundus photographs:

$$\left.\text{Wartery}=0.88\times \sqrt{(W_{1}^{2}}+W_{2}^{2});\text{ Wvein}=0.95\times \sqrt{(W_{1}^{2}}+W_{2}^{2})\right.$$

CRAE and CRVE represent the estimated central retinal arteriolar and venular calibers, respectively, derived from the six largest arterioles and venules in the peripapillary region. The artery-to-vein ratio (AVR) was defined as the ratio of CRAE to CRVE, providing an overall measure of retinal vessel caliber balance (Knudtson et al., 2003).

### 2.3 Statistical analysis

Statistical analyses were performed using R software (version 4.4.1; R Foundation for Statistical Computing, Vienna, Austria). Continuous variables are presented as mean ± standard deviation (SD), and categorical variables as frequencies and percentages. Comparisons among the four myopia severity groups (low, moderate, high, and super-high) were conducted using one-way analysis of variance or the Kruskal–Wallis test. Post hoc pairwise comparisons were conducted using the Bonferroni or Dunn correction when applicable. For regression analyses, myopia severity was dichotomized into non-high (low and moderate) and high (high and super-high) myopia. Univariable logistic regression was first performed to identify candidate parameters, followed by correlation and collinearity assessments. Multivariable logistic regression models were then constructed with adjustment for age and sex. Receiver operating characteristic (ROC) analysis was used to evaluate the discriminative performance of selected parameters. To account for inter-eye correlation among participants with both eyes included, generalized estimating equations (GEE) with an exchangeable correlation structure were used for all logistic and linear regression analyses, clustering by participant ID (Yang et al., 2024). A sensitivity analysis using only the right eye from each participant (n = 274 eyes) was also performed. Linear regression analyses were performed to examine associations between spherical equivalent (SE) and selected parameters from fundus images, with additional adjustment for age and sex. Internal validation of the multivariable model was performed using 10-fold cross-validation and bootstrap resampling with 1,000 iterations. Model calibration was assessed using the Hosmer-Lemeshow goodness-of-fit test. A two-sided P value <0.05 was considered statistically significant.

## 3 Results

### 3.1 Baseline characteristics

The overall mean age of the study population was 25.36 ± 6.36 years. Age varied significantly among groups, showing an increasing trend with greater myopia severity (P < 0.001). Participants in the low myopia group were younger, whereas those in the super-high myopia group tended to be older. Gender distribution differed across groups, with a higher proportion of males in the low and moderate myopia groups and a relatively higher proportion of females in the high and super-high myopia groups. The baseline data of the enrolled patients are shown in Table 1.

**TABLE 1:** Baseline characteristics of the study population.

Variable	All	Low	Moderate	High	Superhigh	Statistic	P value
n (%)	539	166 (30.80%)	194 (35.99%)	131 (24.30%)	48 (0.09%)	​	​
Age	25.16 ± 6.35	22.24 ± 5.67	25.88 ± 6.48	26.58 ± 5.15	30.73 ± 5.86	108.08	0.000
Gender	187 (34.69%)	19 (11.45%)	68 (35.05%)	72 (54.96%)	28 (58.33%)	​	​

### 3.2 Group differences in fundus parameters across myopia severity

Significant differences in nearly all fundus structural and vascular parameters were observed among refractive groups (low, moderate, high, and super-high myopia) (Table 2). Fundus tessellation-related parameters showed marked intergroup differences (all P < 0.001). Mean FT density, total FT area, macular FT area, and peripapillary FT area increased progressively with increasing myopia severity (all P < 0.001). Similarly, peripapillary atrophy–related parameters, including the PPA/D area ratio, peripapillary atrophy width, peripapillary atrophy height, and both horizontal and vertical PPA-to-disc diameter ratios, demonstrated significant intergroup differences (all P < 0.001), with larger values observed in eyes with more severe myopia.

**TABLE 2:** Quantitative analysis of fundus vasculature in different refractive states.

Variable	All	Low	Moderate	high	Superhigh	Statistic	P value
Mean.FT.density	0.12 ± 0.09	0.07 ± 0.05	0.11 ± 0.07	0.17 ± 0.09	0.24 ± 0.09	167.44	0.00
Total.FT.area	73.94 ± 55.13	39.96 ± 30.97	66.74 ± 41.59	99.37 ± 50.66	151.15 ± 71.00	169.81	0.00
Macular.FT.area	3.89 ± 4.31	1.24 ± 1.61	3.07 ± 3.35	5.98 ± 4.34	10.61 ± 4.38	189.88	0.00
Peripapillary.FT.area	3.72 ± 3.19	1.83 ± 1.81	3.36 ± 2.81	5.15 ± 3.15	7.76 ± 3.07	151.24	0.00
PPA/D.area.ratio	0.53 ± 0.65	0.23 ± 0.25	0.45 ± 0.35	0.67 ± 0.46	1.58 ± 1.39	164.18	0.00
Peripapillary.atrophy.width	795.58 ± 1671.84	576.16 ± 1728.36	621.22 ± 506.86	848.18 ± 575.12	2115.60 ± 4177.86	134.90	0.00
Peripapillary.atrophy.height	1444.24 ± 835.61	967.16 ± 765.57	1406.57 ± 697.54	1714.57 ± 562.58	2508.60 ± 944.08	131.26	0.00
Horizontal.PPA–to–disc.diameter.ratio	1.12 ± 0.51	0.89 ± 0.55	1.12 ± 0.46	1.30 ± 0.33	1.58 ± 0.38	107.94	0.00
Vertical.PPA–to–disc.diameter.ratio	1.102 ± 0.508	0.835 ± 0.535	1.095 ± 0.441	1.269 ± 0.314	1.597 ± 0.554	98.76	0.00
HCDR	0.48 ± 0.08	0.49 ± 0.07	0.48 ± 0.07	0.46 ± 0.08	0.47 ± 0.08	3.75	0.01
VCDR	0.42 ± 0.08	0.43 ± 0.08	0.42 ± 0.07	0.39 ± 0.08	0.40 ± 0.09	7.15	0.00
Vertical.optic.disc.diameter	1725.88 ± 181.09	1782.05 ± 167.47	1707.64 ± 162.26	1663.44 ± 171.25	1775.81 ± 248.49	37.91	0.00
Horizontal.optic.disc.diameter	1467.03 ± 211.96	1552.86 ± 194.82	1432.44 ± 195.53	1407.11 ± 203.70	1473.50 ± 261.23	51.42	0.00
Neuroretinal.rim.width	445.18 ± 56.84	453.06 ± 53.74	435.39 ± 49.07	444.03 ± 58.83	460.65 ± 80.70	8.34	0.04
Vertical.optic.cup.diameter	725.04 ± 186.65	779.07 ± 185.09	722.62 ± 176.24	661.63 ± 180.39	721.00 ± 198.58	28.46	0.00
Horizontal.optic.cup.diameter	702.91 ± 178.27	759.96 ± 174.68	692.21 ± 166.85	651.05 ± 177.38	690.38 ± 188.87	32.72	0.00
AVR	0.72 ± 0.07	0.72 ± 0.06	0.72 ± 0.08	0.74 ± 0.07	0.71 ± 0.08	5.90	0.12
d0_a	1.14 ± 0.05	1.15 ± 0.05	1.14 ± 0.04	1.13 ± 0.04	1.11 ± 0.05	20.86	0.00
d1_a	1.08 ± 0.05	1.09 ± 0.05	1.08 ± 0.04	1.08 ± 0.04	1.06 ± 0.06	11.51	0.01
d2_a	1.05 ± 0.05	1.06 ± 0.05	1.06 ± 0.05	1.05 ± 0.04	1.04 ± 0.06	6.91	0.07
sl_a	0.96 ± 0.12	0.98 ± 0.10	0.96 ± 0.13	0.97 ± 0.14	0.90 ± 0.13	15.69	0.00
d0_v	1.11 ± 0.04	1.11 ± 0.04	1.11 ± 0.04	1.10 ± 0.04	1.09 ± 0.05	14.88	0.00
d1_v	1.05 ± 0.05	1.06 ± 0.05	1.05 ± 0.04	1.04 ± 0.05	1.03 ± 0.05	14.88	0.00
d2_v	1.03 ± 0.05	1.03 ± 0.05	1.03 ± 0.05	1.02 ± 0.05	1.01 ± 0.05	13.23	0.00
sl_v	0.95 ± 0.13	0.94 ± 0.12	0.95 ± 0.12	0.96 ± 0.15	0.97 ± 0.11	3.21	0.36
length_avg_a	82.93 ± 14.62	85.86 ± 14.04	81.92 ± 14.49	80.80 ± 15.02	82.68 ± 14.83	11.85	0.01
curvature_avg_a	1.05 ± 0.04	1.05 ± 0.04	1.05 ± 0.04	1.05 ± 0.04	1.04 ± 0.04	6.40	0.09
angle_avg_a	62.58 ± 35.39	65.31 ± 33.72	65.68 ± 33.40	57.77 ± 40.10	53.70 ± 33.26	7.95	0.05
asymmetry_avg_a	26.73 ± 21.99	26.99 ± 19.37	28.46 ± 22.91	26.70 ± 24.53	18.89 ± 17.90	8.22	0.04
brunch_avg_a	1.90 ± 1.08	1.94 ± 0.96	2.04 ± 1.14	1.73 ± 1.11	1.71 ± 1.04	3.40	0.33
length_avg_v	82.66 ± 15.41	81.53 ± 13.94	83.05 ± 16.29	82.30 ± 16.00	86.04 ± 14.83	4.60	0.20
curvature_avg_v	1.05 ± 0.04	1.05 ± 0.04	1.05 ± 0.04	1.05 ± 0.04	1.03 ± 0.03	10.73	0.01
angle_avg_v	63.00 ± 35.54	68.64 ± 27.75	63.97 ± 38.49	56.21 ± 39.19	58.15 ± 34.00	7.19	0.07
asymmetry_avg_v	28.32 ± 22.49	32.00 ± 19.77	27.79 ± 24.12	25.72 ± 24.09	24.86 ± 18.58	9.22	0.03
brunch_avg_v	1.81 ± 1.06	2.10 ± 0.97	1.71 ± 1.04	1.59 ± 1.14	1.75 ± 1.00	28.13	0.00
vessel_length_density	0.01 ± 0.00	0.01 ± 0.00	0.01 ± 0.00	0.01 ± 0.00	0.01 ± 0.00	19.45	0.00
vessel_density	0.14 ± 0.02	0.14 ± 0.02	0.15 ± 0.02	0.15 ± 0.02	0.13 ± 0.02	34.51	0.00

Significant differences were also observed in optic disc morphology. Both horizontal cup-to-disc ratio (HCDR) and vertical cup-to-disc ratio (VCDR) varied across refractive groups (P = 0.011 and P < 0.001, respectively). In addition, optic disc diameters, optic cup diameters, and neuroretinal rim width differed significantly among groups (all P < 0.05).

Regarding vascular parameters quantified from color fundus photographs, the artery-to-vein ratio (AVR) did not differ significantly across groups (P = 0.116). In contrast, several vascular fractal and geometric descriptors showed significant group differences. The fractal dimensions of both arterial and venous networks (d0_a, d1_a, d0_v, d1_v, and d2_v) decreased with increasing myopia severity, indicating reduced vascular complexity in highly myopic eyes. This finding is consistent with previous reports demonstrating that fractal dimension decreases with increasing myopic severity. Selected vascular geometric parameters, including arterial vessel length (length_avg_a), venous curvature (curvature_avg_v), asymmetry_avg_v, and venous branching number (branch_avg_v), also differed significantly among groups. Furthermore, vessel length density and vessel density differed significantly across refractive states (both P < 0.001), with lower values observed in the super-high myopia group.

### 3.3 Logistic regression: associations between parameters and myopia severity grouping

#### 3.3.1 Univariable logistic regression

In univariable logistic regression analyses, multiple fundus-derived structural and vascular parameters were significantly associated with more severe myopia grouping (Table 3). FT-related parameters showed the strongest positive associations. Specifically, macular FT area, total FT area, mean FT density, and peripapillary FT area were all significantly associated with increased odds of high or super-high myopia. Similarly, peripapillary atrophy-related parameters, including peripapillary atrophy height and width, were also positively associated with myopia severity.

**TABLE 3:** Logistic regression analysis of retinal vascular parameters in myopia patients.

Variable	Beta_uni	OR_uni	P_uni	Beta_multi	OR_multi	P_multi
Mean.FT.density	1.147	3.148 (2.575, 3.847)	4.12e-29	​	​	​
Total.FT.area	1.217	3.376 (2.733, 4.170)	1.55e-29	​	​	​
Macular.FT.area	1.297	3.658 (2.975, 4.497)	8.33e-35	1.073	2.925 (2.290, 3.737)	8.54e-18
Peripapillary.FT.area	1.038	2.823 (2.342, 3.404)	1.46e-27	​	​	​
Peripapillary.atrophy.width	1.171	3.225 (1.912, 5.439)	1.13e-05	​	​	​
Peripapillary.atrophy.height	0.966	2.629 (2.157, 3.203)	8.77e-22	0.406	1.501 (1.177, 1.913)	0.00104
HCDR	−0.141	0.869 (0.741, 1.018)	0.0816	​	​	​
VCDR	−0.281	0.755 (0.642, 0.888)	0.000695	​	​	​
Vertical.optic.disc.diameter	−0.342	0.710 (0.598, 0.843)	8.98e-05	​	​	​
Horizontal.optic.disc.diameter	−0.41	0.664 (0.558, 0.790)	3.93e-06	0.073	1.075 (0.817, 1.416)	0.605
Neuroretinal.rim.width	−0.119	0.887 (0.751, 1.048)	0.159	​	​	​
Vertical.optic.cup.diameter	−0.333	0.716 (0.607, 0.845)	7.63e-05	−0.41	0.664 (0.521, 0.846)	0.000939
Horizontal.optic.cup.diameter	−0.309	0.734 (0.621, 0.867)	0.000286	​	​	​
AVR	0.01	1.010 (0.863, 1.183)	0.897	​	​	​
d0_a	−0.438	0.645 (0.547, 0.762)	2.38e-07	−0.152	0.859 (0.692, 1.066)	0.168
d1_a	−0.344	0.709 (0.601, 0.836)	4.46e-05	​	​	​
d2_a	−0.29	0.748 (0.635, 0.882)	0.000552	​	​	​
sl_a	−0.244	0.784 (0.669, 0.918)	0.00259	−0.152	0.859 (0.725, 1.019)	0.0813
d0_v	−0.259	0.772 (0.657, 0.907)	0.0016	0.155	1.168 (0.933, 1.462)	0.175
d1_v	−0.249	0.780 (0.665, 0.915)	0.00224	​	​	​
d2_v	−0.218	0.804 (0.686, 0.943)	0.00728	​	​	​
sl_v	0.145	1.156 (0.987, 1.353)	0.0716	0.028	1.028 (0.864, 1.223)	0.754
length_avg_a	−0.232	0.793 (0.676, 0.931)	0.00467	−0.274	0.760 (0.614, 0.941)	0.0117
curvature_avg_a	−0.129	0.879 (0.748, 1.033)	0.116	​	​	​
angle_avg_a	−0.186	0.831 (0.708, 0.974)	0.0222	−0.207	0.813 (0.665, 0.995)	0.0443
asymmetry_avg_a	−0.081	0.922 (0.787, 1.080)	0.315	​	​	​
brunch_avg_a	−0.126	0.882 (0.753, 1.032)	0.118	​	​	​
length_avg_v	0.055	1.057 (0.904, 1.235)	0.488	​	​	​
curvature_avg_v	−0.165	0.848 (0.723, 0.994)	0.0423	−0.082	0.921 (0.775, 1.095)	0.352
angle_avg_v	−0.147	0.863 (0.738, 1.010)	0.0657	0.012	1.012 (0.767, 1.335)	0.933
asymmetry_avg_v	−0.17	0.844 (0.721, 0.987)	0.0338	−0.094	0.910 (0.728, 1.137)	0.406
brunch_avg_v	−0.206	0.814 (0.692, 0.957)	0.0129	−0.087	0.916 (0.702, 1.197)	0.521
vessel_length_density	−0.223	0.800 (0.678, 0.945)	0.00847	​	​	​
vessel_density	−0.279	0.756 (0.638, 0.896)	0.00124	−0.145	0.865 (0.640, 1.170)	0.347

In contrast, several optic disc and retinal vascular parameters showed inverse associations. VCDR, optic disc diameters, optic cup diameters, and multiple vascular fractal parameters (including d0_a, d1_a, d2_a, d0_v, d1_v, and d2_v) were negatively associated with severe myopia. In addition, vessel density and vessel_length_density were significantly lower in high or super-high myopia eyes. Several vascular geometric parameters were also associated with high myopia risk in univariable analyses, including length_avg_a, angle_avg_a, curvature_avg_v, and venous branching parameters.

#### 3.3.2 Correlation analysis and collinearity assessment

**FIGURE 2:**
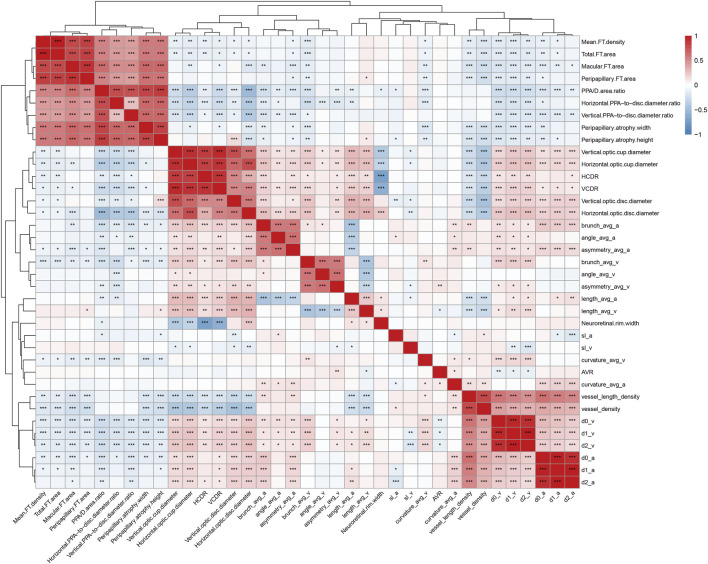
Spearman correlation heatmap showing the relationships among retinal vascular parameters derived from color fundus photography. The color scale represents the Spearman’s rank correlation coefficient, with red indicating positive correlations and blue indicating negative correlations. Deeper colors represent stronger correlations. Asterisks indicate statistically significant correlations (*p < 0.05, **p < 0.01, ***p < 0.001).

Correlation analysis revealed strong interrelationships among several candidate predictors (Figure 2). FT-related parameters were highly correlated with each other, with near-perfect correlation observed between mean FT density and total FT area, and strong correlations with macular FT area and peripapillary FT area. High correlations were also present among arterial and venous fractal dimension parameters, as well as optic cup-to-disc descriptors, indicating potential redundancy among variables representing similar anatomical features.

Variance inflation factor (VIF) analysis demonstrated acceptable VIF values for all selected predictors (all VIF <5), indicating no severe multicollinearity. Based on these findings, representative and clinically interpretable parameters were selected for inclusion in the multivariable logistic regression model.

#### 3.3.3 Multivariable logistic regression

After adjustment for age and sex, multivariable logistic regression identified several independent factors associated with myopia severity grouping (Table 3). Among structural parameters, macular FT area remained the strongest independent factor (OR = 2.925, 95% CI 2.290–3.737, P < 0.001). In addition, peripapillary atrophy height retained a significant positive association (OR = 1.501, 95% CI 1.177–1.913, P = 0.001). Vertical optic cup diameter showed an independent inverse association with myopia severity (OR = 0.664, 95% CI 0.521–0.846, P < 0.001). The GEE results were consistent with standard logistic regression. Sensitivity analysis using only the right eye from each participant yielded similar results (macular FT area OR = 2.91, P < 0.001; combined model AUC = 0.864).

Regarding vascular parameters quantified from color fundus photographs, length_avg_a and angle_avg_a showed inverse associations with high myopia (OR = 0.760 and OR = 0.813, respectively), indicating that shorter vessel length and smaller branching angles were associated with increased odds of high myopia. In contrast, several vascular density and fractal parameters that were significant in the univariable analyses did not retain statistical significance after multivariable adjustment.

### 3.4 Discriminative performance of parameters for myopia severity

**FIGURE 3:**
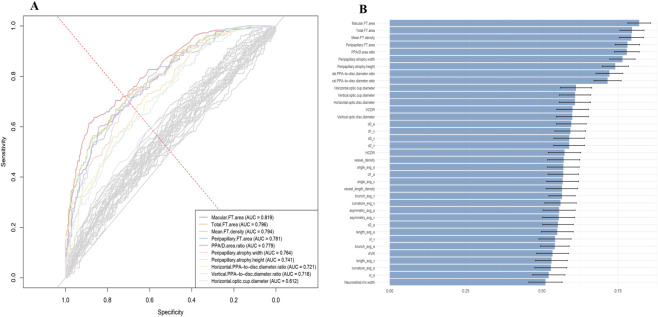
ROC curve analysis for the discriminative ability of retinal vascular parameters. The true positive rate (sensitivity) is plotted against the false positive rate (1-specificity) for each parameter **(A)** ROC curves of the selected retinal vascular parameters. The area under the curve (AUC) for each parameter is displayed in the legend of panel B** (B)** Forest plot showing the AUCs with their corresponding 95% confidence intervals (CIs) for each retinal vascular parameter.

Among individual parameters, FT-related metrics demonstrated the strongest discriminative performance in ROC analyses (Figure 3). Macular FT area showed the highest AUC (0.819, 95% CI 0.782–0.857), followed by total FT area, mean FT density, and peripapillary FT area (AUCs ranging from 0.781 to 0.796). Peripapillary atrophy-related indices exhibited moderate discriminative ability (AUCs 0.741–0.779). In contrast, vascular density, vascular geometry/fractal parameters, and optic disc or cup descriptors generally showed limited discriminative performance, with AUCs close to 0.60.

**FIGURE 4:**
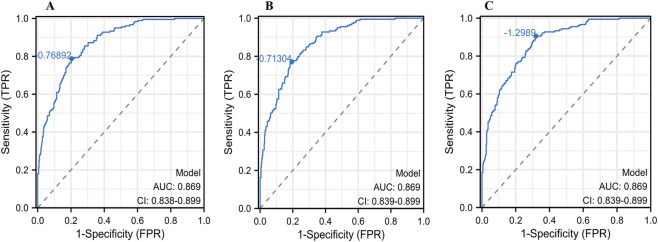
Receiver operating characteristic (ROC) curves for combined discriminative models **(A)** Model A includes age, sex, and macular FT area, yielding an AUC of 0.819 (95% CI 0.782–0.857) **(B)** Model B adds peripapillary atrophy height and vertical optic cup diameter to the variables in Model A, yielding an AUC of 0.869 (95% CI 0.838–0.899) **(C)** Model C further adds arterial vessel length (length_avg_a) and arterial branching angle (angle_avg_a) to the variables in Model B, yielding an AUC of 0.869 (95% CI 0.839–0.899), which is nearly identical to that of Model **(B)**. The overlap between the ROC curves for Models B and C indicates that the addition of vascular parameters did not materially improve discriminative performance beyond the structural parameters already included in Model **(B)**. The diagonal gray line represents the reference line (AUC = 0.5).

A combined discriminative model incorporating age, sex, macular FT area, peripapillary atrophy height, and vertical optic cup diameter achieved an AUC of 0.869 (95% CI 0.838–0.899) (Figure 4). The addition of the arterial geometric parameter length_avg_a did not further improve the AUC (AUC = 0.869, 95% CI 0.839–0.899). Similarly, additional inclusion of angle_avg_a yielded a comparable AUC (0.869, 95% CI 0.839–0.899), indicating that structural parameters predominantly accounted for the discriminative performance.

To assess the robustness of the combined model (macular FT area, PPA height, and vertical optic cup diameter, adjusted for age and sex), we performed 10-fold cross-validation. The cross-validated AUC was 0.857 (95% CI 0.828–0.886), which is only slightly lower than the original AUC of 0.869, indicating minimal overfitting. Bootstrap resampling with 1,000 iterations was also performed to estimate the optimism-corrected AUC, which yielded a corrected AUC of 0.861 (95% CI 0.832–0.890). Additionally, we assessed model calibration using the Hosmer-Lemeshow goodness-of-fit test, which yielded a P-value of 0.32, indicating no significant difference between predicted and observed risks (good calibration). These validation analyses support the robustness of the model, although external validation in an independent cohort is needed before clinical implementation.

### 3.5 Linear associations between parameters and spherical equivalent

To further explore whether key parameters exhibited progressive changes with myopia severity, linear regression analyses were performed using spherical equivalent (SE) as a continuous variable (Table 4). In univariable analyses, macular FT area showed a strong negative association with SE (β = −0.836, P < 0.001), indicating a marked increase in macular FT area with increasing myopia severity (i.e., more negative SE). This association remained robust after adjustment for age and sex (β = −0.797, standardized β = −0.642, P < 0.001). Similarly, length_avg_a demonstrated a significant positive association with SE in univariable analysis (β = 0.414, P = 0.023), indicating that shorter arterial vessel lengths were associated with more severe myopia. After adjusting for age and sex, SE remained independently associated with length_avg_a (β = 0.536, standardized β = 0.127, P = 0.008). Age and sex were not independently associated with length_avg_a in the multivariable model. These findings indicate that both structural and selected vascular parameters change progressively with increasing myopia severity, supporting their potential role as early biomarkers of myopic fundus alterations.

**TABLE 4:** Line regression analysis of retinal vascular parameters in myopia patients.

Variable	Univariate analysis		Multivariate analysis*		
	Unstandardized β	P value	Unstandardized β	Standardized β	P value
Macular FT	−0.836	0.000	−0.797	−0.642	0.000
Length_A	0.414	0.023	0.536	0.127	0.008

*Multivariate regression model was adjusted for age and sex.

## 4 Discussion

This systematic analysis of 539 eyes with varying degrees of myopia revealed progressive alterations in fundus tessellation (FT), optic disc morphology, and retinal vascular parameters, all quantified from standard color fundus photographs using AI-assisted techniques. The key findings were: (1) macular FT area was the strongest independent factor discriminating high/super-high myopia from low-to-moderate myopia (OR = 2.925); (2) peripapillary atrophy (PPA) parameters were significantly enlarged in highly myopic eyes; (3) fractal dimensions of retinal vascular networks decreased with advancing myopia; (4) arterial vessel length and branching angle showed inverse associations with spherical equivalent; and (5) a multi-parameter model combining macular FT area, PPA height, and vertical cup diameter achieved excellent discriminative performance (AUC = 0.869). These findings provide quantitative evidence for characterizing myopia-related fundus alterations using widely available color fundus photography.

### 4.1 Fundus tessellation as an imaging biomarker of myopia severity

Macular FT area demonstrated the strongest discriminative performance (AUC = 0.819) among all parameters. This finding aligns closely with recent quantitative studies using deep learning approaches. In a study of children with early-onset high myopia (mean age ≤6 years, mean SE -9.35 D), FT density was significantly correlated with axial length (r = 0.46, p = 1.47 × 10^−3^) (Guo et al., 2026). Multivariable regression analysis identified FT density in the 6-mm nasal and superonasal quadrants as independent predictors of axial length, highlighting the importance of regional FT assessment. Their study suggests that quantitative FT assessment sensitively may reflects posterior pole structural alterations even in young children, and our results extend this finding to older populations (mean age 25.36 ± 6.36 years), indicating that the value of FT as a myopia biomarker spans from childhood through adulthood.

The superior performance of macular over peripapillary FT area may reflect regional biomechanical differences. The macula, located at the central posterior pole, experiences greater tensile strain during axial elongation compared to the peripapillary region, where tissues may be partially anchored by the scleral canal and optic nerve sheath. Additionally, the macular choroid is physiologically thinner, and the retinal pigment epithelium (RPE) has higher metabolic demand, rendering this region more vulnerable to stretching-induced changes (Venkatesh et al., 2026). This interpretation is supported by Huang et al. (2023), who found stronger correlations between macular FT and spherical equivalent than for peripapillary FT.

Regarding methodology, compared with alternative AI-based FT quantification methods such as texture-based feature extraction or standard U-Net architectures, the HR-Net used in our study preserves higher spatial resolution, which is advantageous for segmenting subtle FT boundaries (Guo et al., 2024; Cao et al., 2025). Our use of manual review with minor corrections (applied in 12.5% of images) enhanced accuracy while maintaining practical throughput.

From a pathophysiological perspective, though not directly tested in this study but FT formation is thought to relate to choroidal thinning and RPE hypopigmentation secondary to axial elongation (Venkatesh et al., 2026). The progressive increase in FT density we observed is consistent with this mechanism. The concept of “fundus refraction offset” (FRO) further supports this interpretation: a more negative FRO has been independently associated with lower macular thickness and decreased choroidal vascularity index (Yii et al., 2025a; Yii et al., 2025b; Zhang et al., 2025a). It is worth noting that age-related choroidal thinning could theoretically contribute to increased FT visibility; however, our statistical models adjusted for age, and the narrow age range of our population minimizes confounding from age-related degenerative processes.

The clinical utility of FT should be understood as a structural biomarker of posterior segment remodeling rather than a substitute for refractive assessment. Two eyes with the same spherical equivalent may have different degrees of choroidal thinning and FT visibility, and this added information may help identify eyes with more advanced structural remodeling. However, because our study is cross-sectional, we cannot claim that FT predicts future complications such as myopic maculopathy or retinal detachment. Longitudinal studies are needed to establish whether baseline FT area is associated with incident complications.

### 4.2 Progressive reduction in retinal vascular network complexity from color fundus photographs

Fractal dimension, as a global descriptor of vascular network complexity, quantifies the space-filling capacity and branching pattern of the retinal microvasculature (Gao et al., 2023). Reduced FD implies simplification of the branching pattern and diminished space-filling capacity (Jonas et al., 2021). In highly myopic eyes, pathological axial elongation likely exerts mechanical traction on the retinal vascular network, leading to vessel straightening, reduced branching, and consequently decreased FD (He et al., 2024; Liu et al., 2021).

Previous studies using ultra-widefield imaging have reported similar findings. In a study of 317 high myopia eyes classified according to the META-PM classification, patients with high myopia had significantly smaller fractal dimensions (1.383 ± 0.060 vs. 1.424 ± 0.038 in healthy controls), reduced vessel density (2.57% ± 0.96% vs. 3.92% ± 0.93%), and fewer vascular branches (201.87 ± 75.92 vs. 271.31 ± 67.37) (Mao et al., 2023). The loss of statistical significance for fractal dimensions after multivariable adjustment, while vessel length (length_avg_a) and branching angle (angle_avg_a) remained independently associated, warrants careful interpretation. Mechanical axial stretching likely directly affects specific geometric parameters, such as straightening vessels (reduced tortuosity) and reducing branching angles, and the observed reduction in global fractal dimension may be a consequence of these individual geometric changes (He et al., 2024; Liu et al., 2021). Our correlation analysis (Figure 2) revealed strong interrelationships among vascular parameters, indicating that collinearity likely contributes to the attenuation of fractal dimensions in the multivariable model. The persistence of length and angle parameters after adjustment suggests that these features may be more direct indicators of mechanical traction on the retinal vasculature, whereas global complexity indices may be more susceptible to confounding by other factors such as image quality or variations in vascular segmentation.

The mechanistic explanations offered here are speculative and based on prior studies. Arteries and veins were analyzed separately because they may respond differently to mechanical stretching: arteries have thicker muscular walls, whereas veins are more distensible and could show earlier geometric alterations (Mao et al., 2023; Gao et al., 2023). Regarding methodology, the box-counting method applied to skeletonized vessel centerlines discards vessel caliber information; alternative methods such as multifractal analysis may provide additional information and represent a direction for future research.

It is important to note that while vascular parameters did not substantially improve the AUC beyond structural parameters, several vascular metrics remained independently associated with myopia severity, suggesting they capture aspects of vascular remodeling not fully explained by structural changes alone.

### 4.3 Clinical implications for risk stratification and comparison with OCT

Our findings carry important clinical implications, particularly regarding the potential for risk stratification using widely available color fundus photography. The strong discriminative performance of macular FT area (AUC = 0.819) suggests that this parameter alone could serve as a screening tool for identifying individuals at high risk of pathological myopia. This is particularly relevant in primary care and community screening settings where access to advanced imaging may be limited. Furthermore, the linear association between macular FT area and spherical equivalent (β = −0.797 after adjustment) indicates that this parameter captures progressive structural changes along the myopia continuum, not merely a binary distinction between high and low myopia. External validation in an independent cohort is needed before clinical implementation of this model.

For clinical implementation, a practical cutoff value for macular FT area would need to be derived from a separate validation cohort with prespecified sensitivity and specificity targets. Based on the distribution of FT areas across our severity groups for illustrative purposes, a lower cutoff might be appropriate for school-based screening programs where high sensitivity is prioritized to avoid missing at-risk children, whereas a higher cutoff could be used in hospital-based triage where specificity is more important to avoid unnecessary referrals. We emphasize that formal cutoff determination requires a separate prospective validation cohort with prespecified sensitivity and specificity targets, as well as consideration of the prevalence of high myopia in the target population. The choice of cutoff should also consider local healthcare resources and the availability of confirmatory testing such as OCT or biometry. Similar findings were reported by Wang et al. (Wang et al., 2026), while their model focused on predicting future progression in children, our cross-sectional model identifies current high myopia status.

Compared with OCT-based parameters, fundus photography offers distinct advantages for large-scale applications. OCT enables visualization of deeper vascular plexuses and provides quantitative metrics such as choroidal thickness and choroidal vascularity index (Chen N et al., 2025; Si et al., 2024). Increased FT density correlates strongly with reduced choroidal thickness (r = −0.48 to −0.61) and decreased choroidal vascularity index (Chen et al., 2023; Guo et al., 2024). Thus, in settings where OCT is unavailable, macular FT area may serve as a practical surrogate for choroidal thinning. However, OCT and fundus photography provide complementary information, and future studies may benefit from integrating both modalities.

However, color fundus photography offers distinct advantages for large-scale screening applications. The technology is mature, standardized, and requires minimal patient cooperation. Recent advances in deep learning have enabled accurate segmentation of retinal vessels and quantification of fractal dimensions from standard fundus photographs, achieving performance comparable to that of OCT angiography for certain parameters (Go et al., 2025; Zekavat et al., 2022). The accuracy of these deep learning systems for vessel segmentation has been reported to exceed 98%, supporting their clinical utility (Zhang et al., 2025b).

Nevertheless, OCT and color fundus photography provide complementary information. While fundus photography captures the *en face* projection of the retinal vasculature, OCT provides depth-resolved information about individual retinal layers and the choroid (Xu et al., 2025; Hu et al., 2025). Future studies may benefit from integrating both modalities to develop more comprehensive risk prediction models.

### 4.4 Study limitations and future directions

Several limitations of this study should be acknowledged. First, the cross-sectional design precludes causal inference. Second, axial length was not measured; our findings should be interpreted as associations with spherical equivalent rather than direct measurements of axial elongation. Third, ocular magnification effects may influence absolute measurements; lack of individual correction may overestimate area-based parameters in highly myopic eyes. Fourth, the single-center design and predominantly young adult population (mean age 25.36 years) may limit generalizability to older adults or other ethnic groups. Fifth, patients with pathologic myopia complications were excluded, so results apply only to uncomplicated simple myopia. Sixth, pre-existing vascular traits could theoretically predispose to myopia progression rather than vascular changes being purely secondary. Finally, while our AI segmentation models achieved high accuracy (Dice = 0.87 for FT, 0.91 for vessels), manual corrections were still required in some cases, indicating room for further improvement. Furthermore, no spherical-to-planar distortion correction was applied to area measurements. Although projection errors in fundus imaging have been well documented (Doelemeyer and Petrig, 2000), our comparative analyses across myopia severity groups remain valid because systematic overestimation in high myopia eyes would not reverse the direction of association. Nevertheless, future studies incorporating axial length-based magnification correction (e.g., using the Bennett formula) or algorithms such as ReLAC for optic disc tilt correction could provide more precise absolute area quantification (Munthuli et al., 2025).

The findings of this study open several avenues for future research. Morphological characterization of FT patch size distribution using connected component analysis emerges as a particularly promising direction. While our study quantified total FT area as a global metric, analyzing the quantity and size distribution of individual FT patches, for example, by categorizing them into small, medium, and large components, could reveal more granular structure-severity correlations. Specifically, diffuse fine tessellation may reflect early retinal pigment epithelium changes, whereas coarse large-area patches might indicate more advanced choroidal thinning.

We therefore propose that future studies apply connected component analysis to investigate whether specific patch size categories are independently associated with myopia severity or predict progression to myopic maculopathy. Building on this key priority, we outline five additional critical areas for future investigation. First, longitudinal cohort studies are needed to validate the prospective value of the imaging parameters identified in this study for predicting high myopia development and progression. Second, integration of fundus photography-derived parameters with OCT-based metrics (e.g., choroidal thickness, choroidal vascularity index) may enable more accurate risk stratification. Third, the application of these AI-assisted quantification techniques to ultra-widefield fundus photography may reveal peripheral retinal changes that are not captured by standard 45° images. Fourth, exploration of associations between imaging parameters and molecular biomarkers (e.g., aqueous humor cytokine levels) could provide mechanistic insights into the pathophysiology of myopia-related vascular remodeling. Fifth, assessment of these parameters in response to myopia control interventions (e.g., low-dose atropine, orthokeratology, peripheral defocus spectacles) may identify imaging biomarkers that predict treatment response.

### 4.5 Conclusions

This study systematically delineated the progressive alterations in fundus tessellation, optic disc morphology, and retinal vascular networks across varying degrees of myopia using AI-assisted quantitative analysis of standard color fundus photographs. Macular fundus tessellation area emerged as the strongest independent factor discriminating high myopia from low-to-moderate myopia. A multi-parameter model integrating macular FT area, peripapillary atrophy height, and vertical optic cup diameter achieved excellent discriminative performance. The progressive reduction in retinal vascular fractal dimensions and geometric parameters suggests vascular network simplification in highly myopic eyes. These findings provide potential imaging biomarkers for myopia risk stratification that can be derived from widely available color fundus photography, supporting their utility in large-scale screening and early intervention programs.

## Data Availability

The original contributions presented in the study are included in the article/supplementary material, further inquiries can be directed to the corresponding authors.
